# Tincture of Thapsus

**Published:** 1879-02

**Authors:** H. G. Piffard

**Affiliations:** New York


### ARTICLE XIV.

*To the Editors of the Chicago Medical Journal and Examiner:*

GENTLEMEN:—In the January number of your journal, Dr. Tucker calls attention to "Tincture of Thapsus" (*sic*), and states that "it has long been a favorite remedy with the so-called 'eclectics,' whose leading pharmacist first called my attention to it." The doctor has fallen into error as regards the name of the plant whose virtues were well known long ago in Europe and even in this country before the revolution. It was formerly called the *thlaspi bursæ pastoris*, but is now named by botanists *capsella bursæ pastoris*, the English name of which is "shepherd's purse." The *Cincinnati Medical Gazette* for Nov., 1878, a journal which appears to be an organ of the "Physio-Medical" sect, contains the following:—"CAPSELLA BURSA PASTORIS.—Some eighteen months ago, Dr. Rademacher, of Germany, spoke highly of this plant as a remedy for irritable bladder," etc.

The *Chicago Medical Times* for Jan., 1879 (an eclectic journal), contains an article on capsella bursa pastoris, illustrated with a wood-cut. In the article we find that: "Dr. Carl Kissel states that, 'it is a vascular remedy, healing affections which appear as chronic diarrhœa, inflammation of the kidneys with sediment of uric acid,'" etc.

The foregoing quotations exhibit a curious mingling of fact and error, and as I have personally used the drug with satisfaction for some time past, I will endeavor to clear up the confusion a little. Under the name Thlaspi burs. past., SCHOEPF (*Materia Medica Americana*, Erlangen, 1787, p. 103) speaks of it as astringent, and useful in hæmorrhages. In Europe it had long been known as a domestic remedy, but Rademacher was the first to give it prominence. In his curious work entitled, "*Rechtfertigung der von den Gelehrten misskaunten verstandesrechten Erfahrungsheillehre der alten Scheidekünstigen Geheimärtze*," —2 vols., Berlin, 1846–7—he speaks of the "Herba Bursæ pastoris" as follows: "*Dieses Kraut galt in der alten Welt für*

*eine blutstillende Arzenei.*" Dr. Carl Kissel was one of Rademacher's followers, and speaks of the drug in his work entitled "Die Heilmittel Rademachers und der naturwissenschaftlichen Therapie," Giessen 1859. My own attention was first called to it by my friend Dr. Frankel of this city, who referred me for further information to the work last mentined. I have used it with satisfaction in cases of irritated bladder in doses of 10 to 30 drops of the tincture sold in homœopathic pharmacies under the name of "Thlaspi." If it should come into frequent use, I would suggest that the old pharmaceutical name, *tinct. bursæ pastoris*, be retained. Respectfully yours,

H. G. PIFFARD, M.D.

NEW YORK, Jan. 20, 1879.

---

FATAL POISONING BY OIL OF CHENOPODIUM.—(*Maryland Medical Journal*, Nov., 1878.)—Dr. T. R. Brown reports the case of a hypochondriac, who imagined himself to be suffering, among other difficulties, from intestinal worms. For this the doctor recommended an ounce of castor oil with twenty drops of turpentine. The patient procured an *ounce and a half* of wormseed oil and added *thirty* drops of turpentine. Within a few hours he acted strangely, his gait being unsteady, face pale and anxious, etc. Following this he sank into a heavy slumber, vomited during sleep, was aroused without much difficulty, but sank into the same sleep again unless constantly diverted. Within two days he became very deaf to sounds of the voice, but acutely sensitive to noises and jars about the house. This was succeeded by an aphasic attack, coming on slowly, and increasing; and this by paralysis of the right arm, pyrexia, unilateral convulsions (right side), involuntary dejections, icterus, general opisthotonic convulsions, coma and death on the fifth day.

No *post-mortem* was allowed. So far as the author knows this is first case of fatal poisoning by this drug on record, though fatal cases have happened before.

